# Supplementary figures and images for: A species-specific lncRNA modulates the reproductive ability of the asian tiger mosquito
Source: Front Bioeng Biotechnol. 2022 Aug 24;10:885767. doi: 10.3389/fbioe.2022.885767 (PMC9448860; doi:10.3389/fbioe.2022.885767)

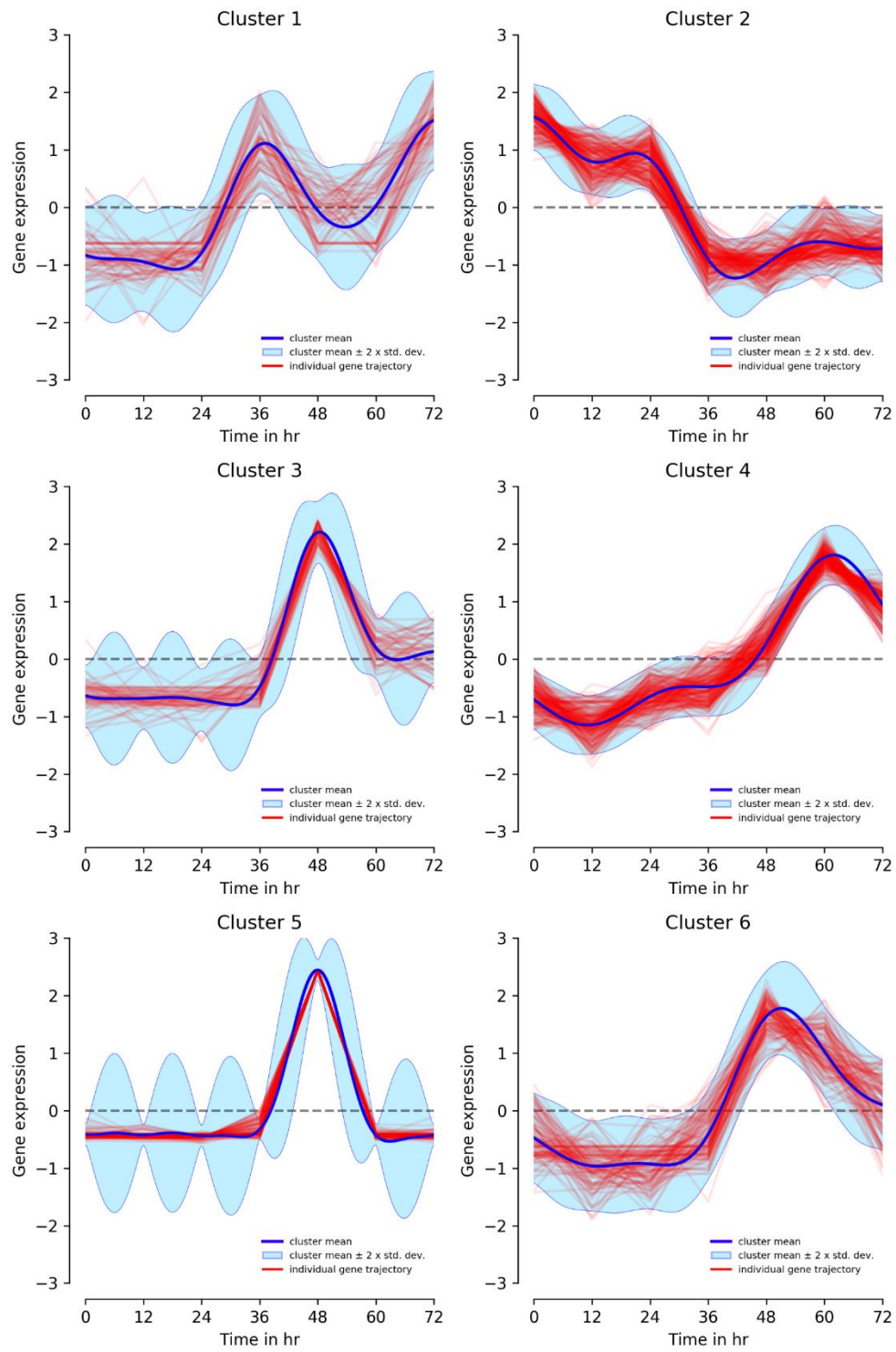

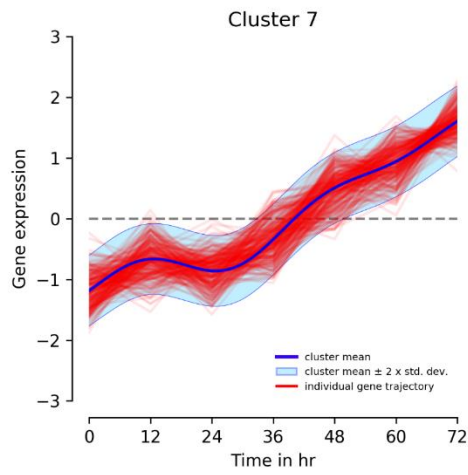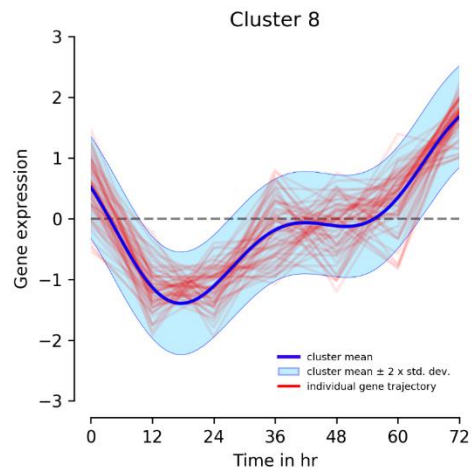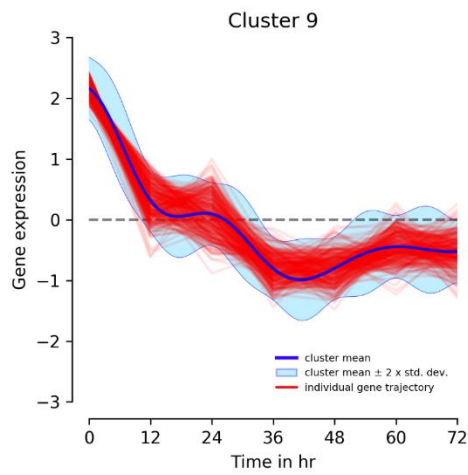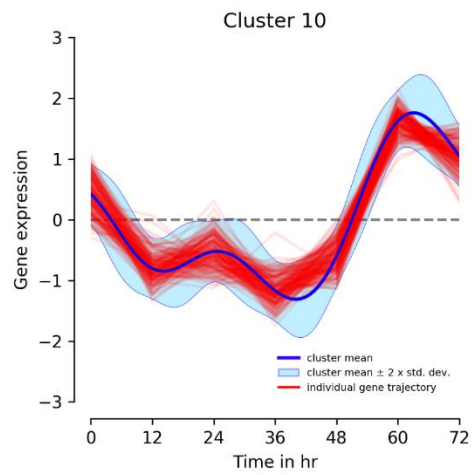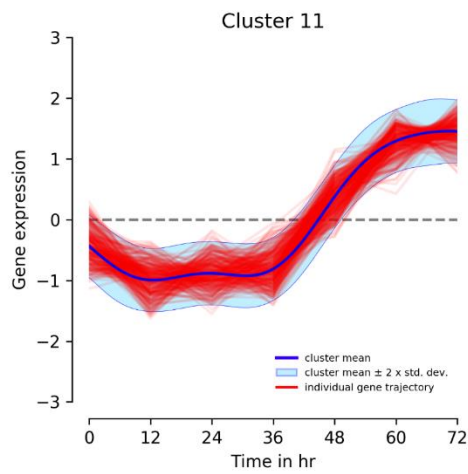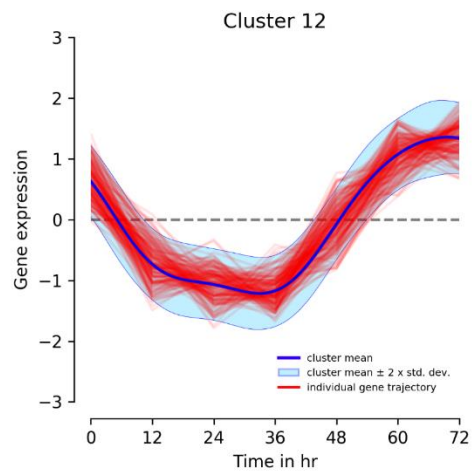

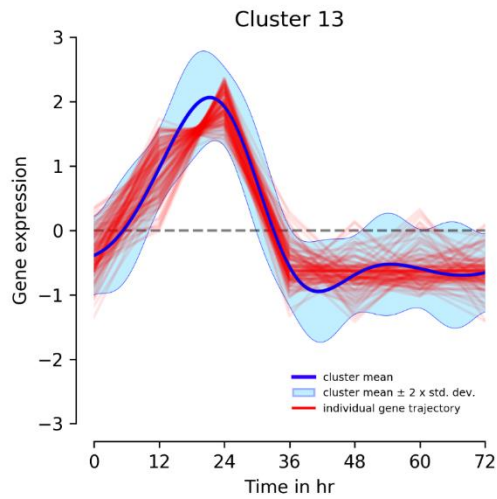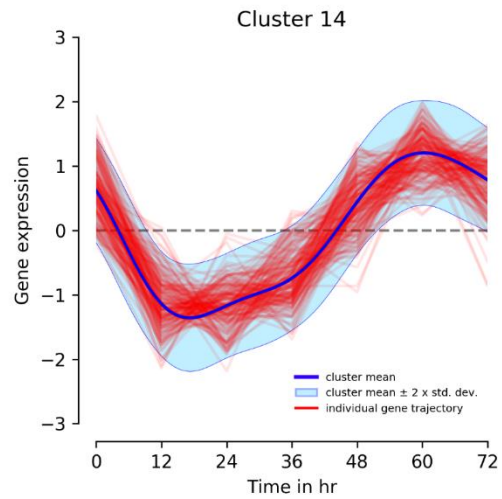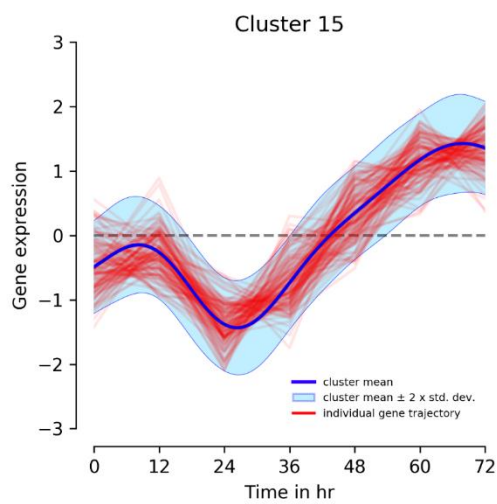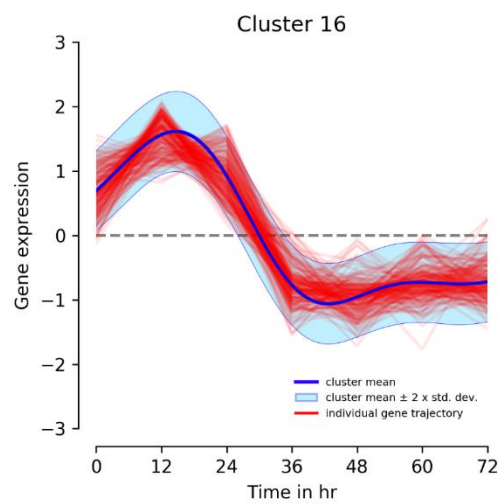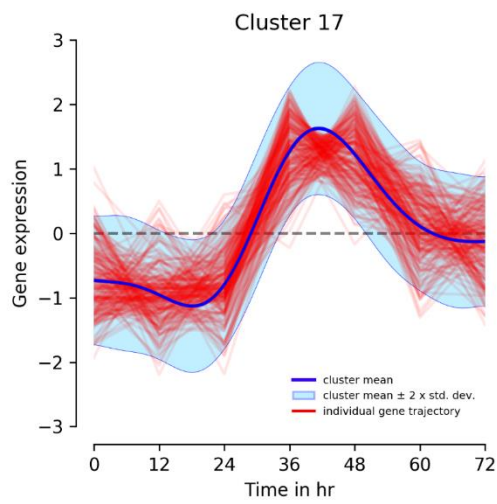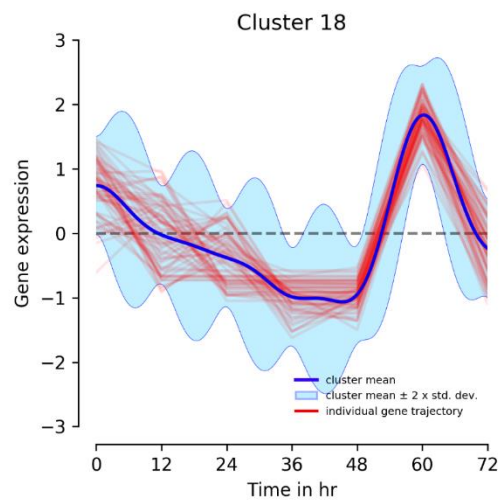

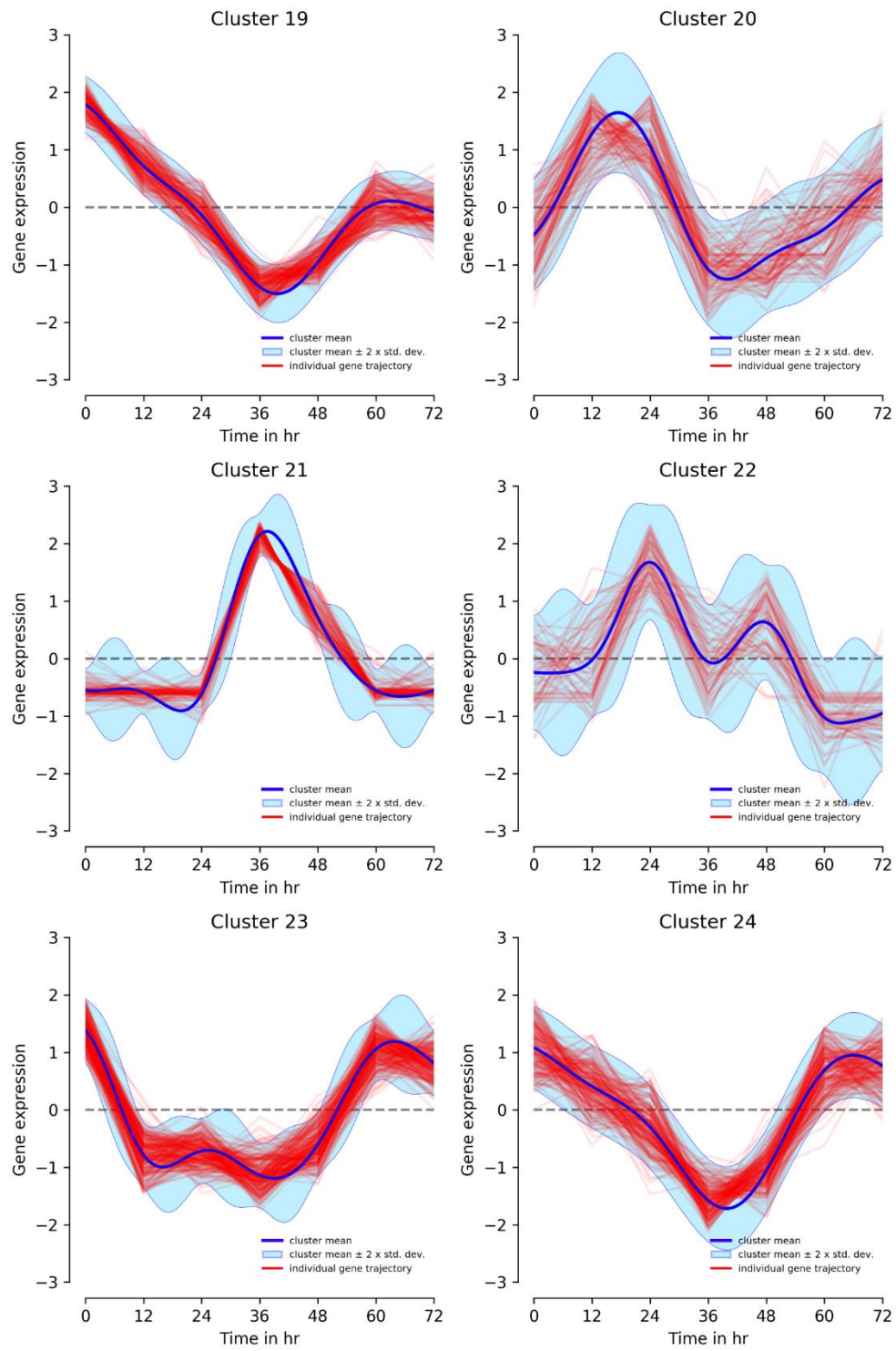

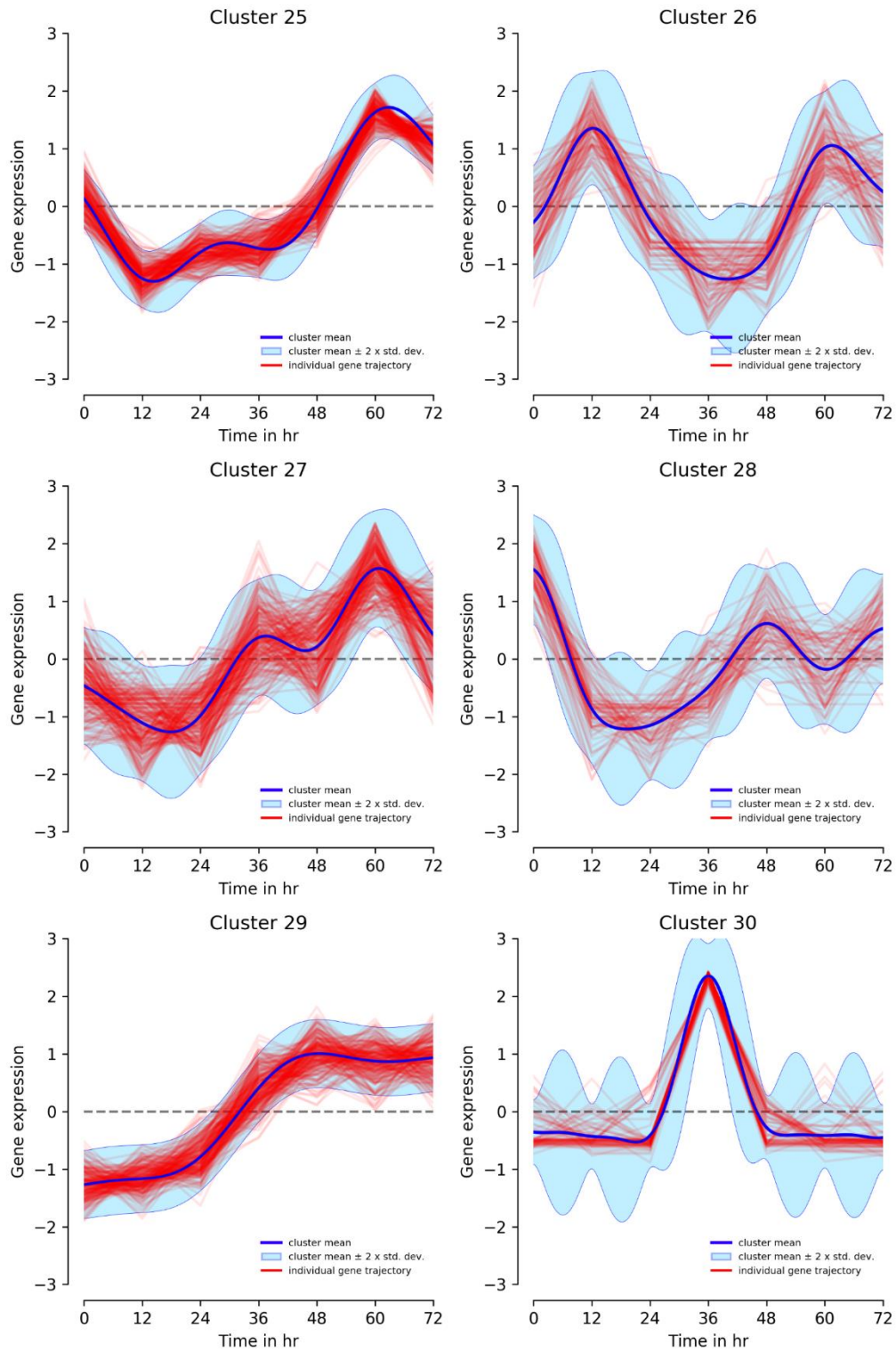

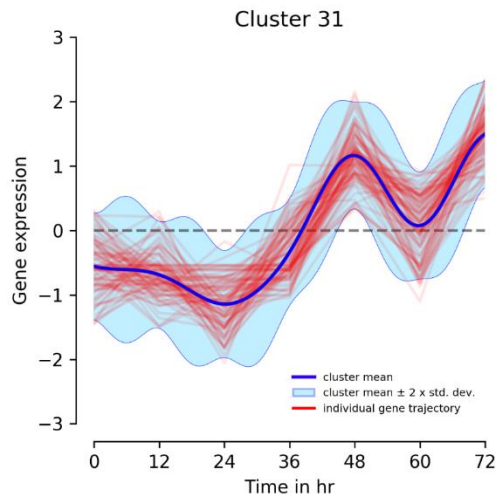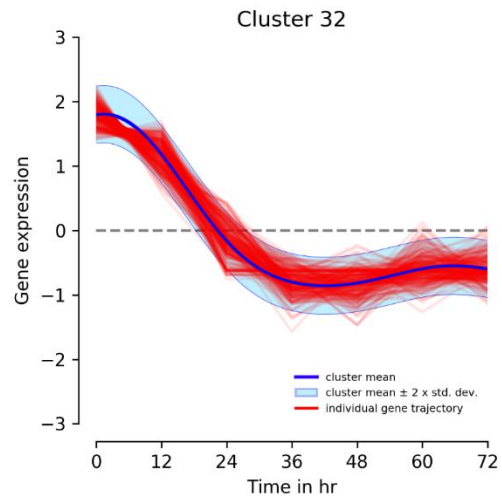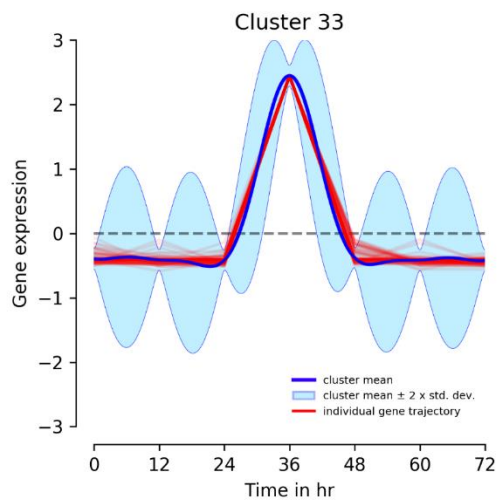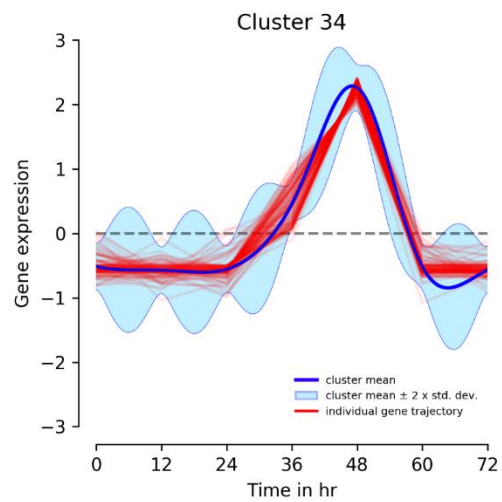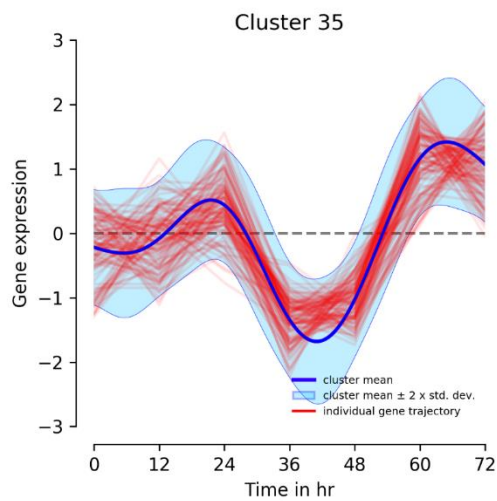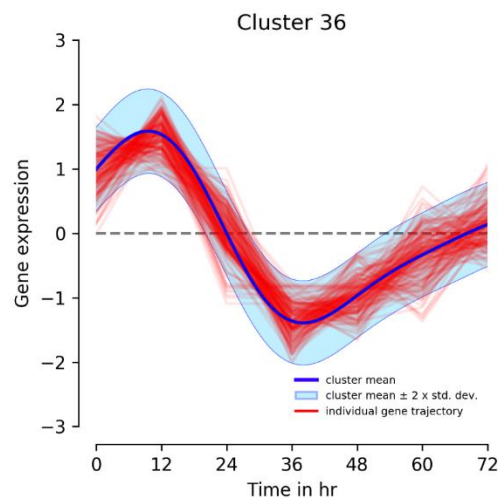

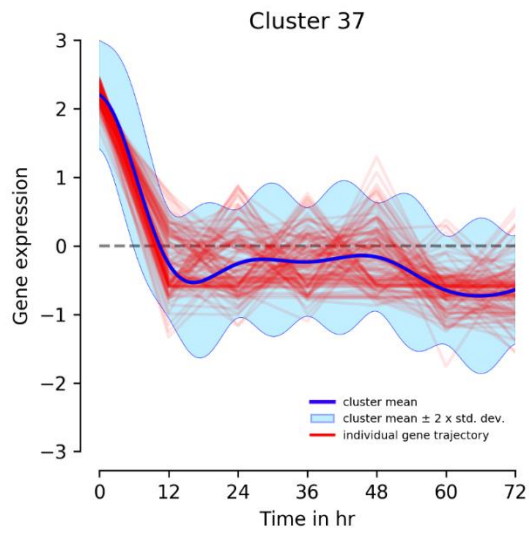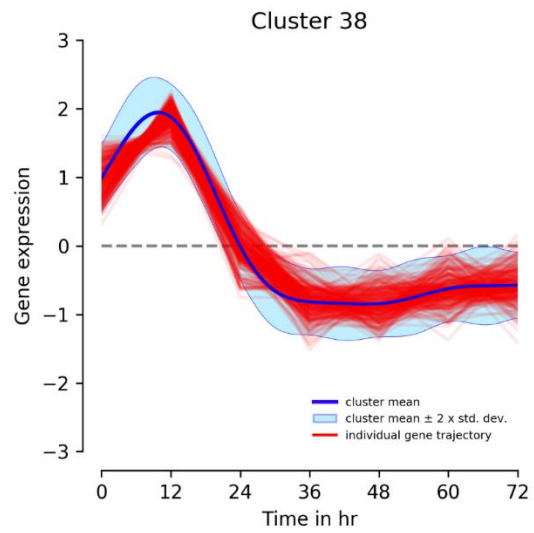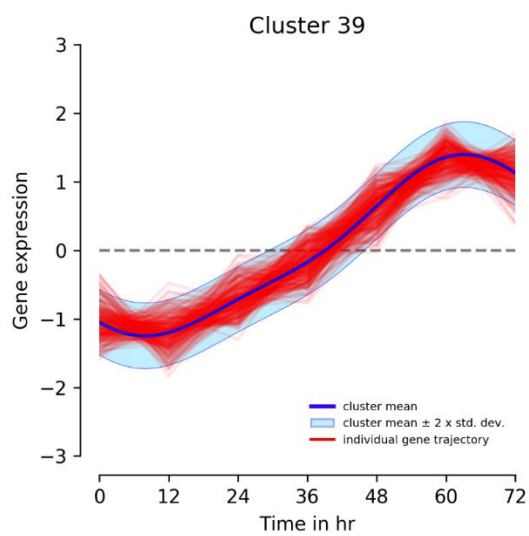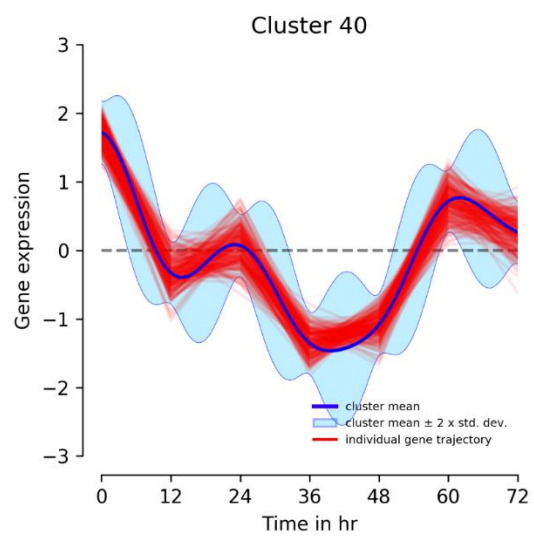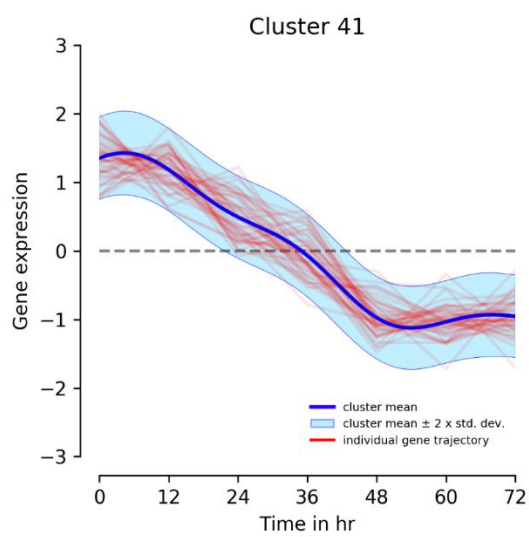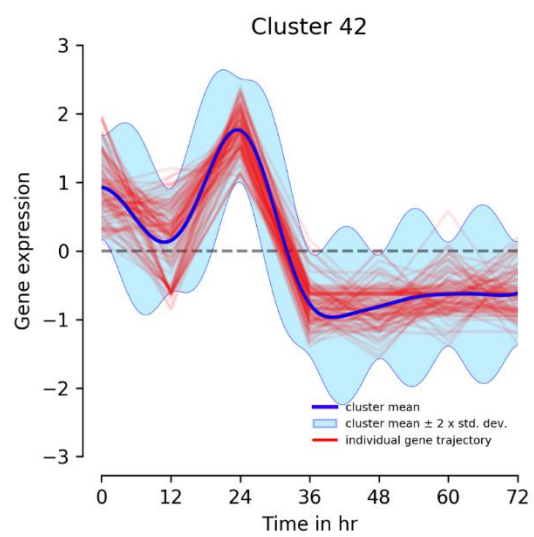

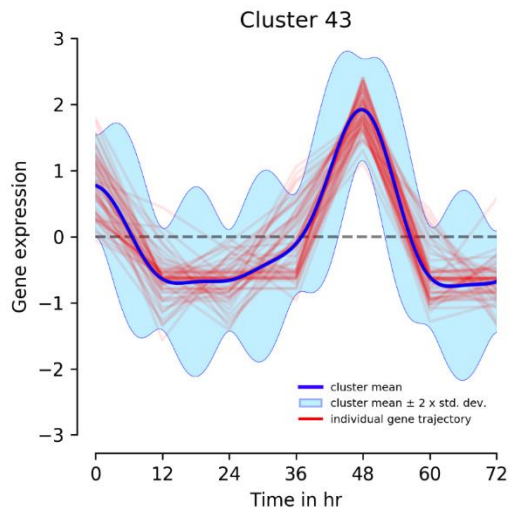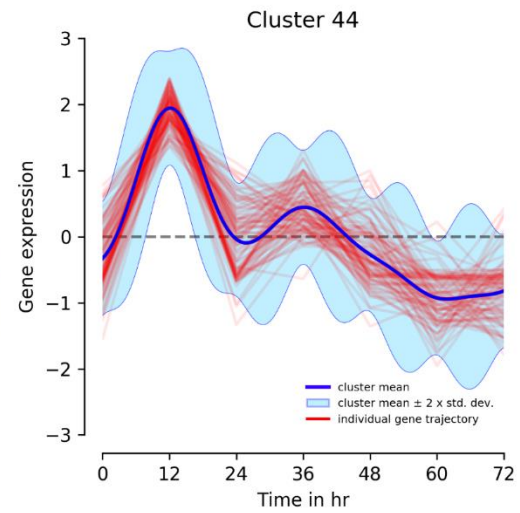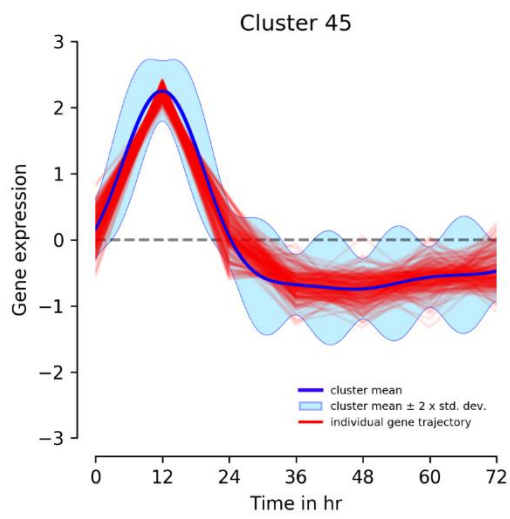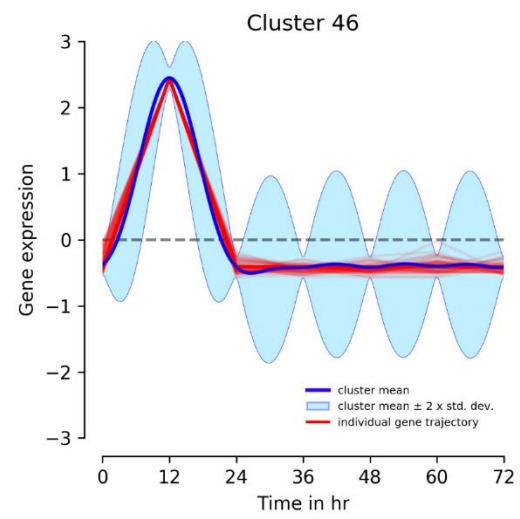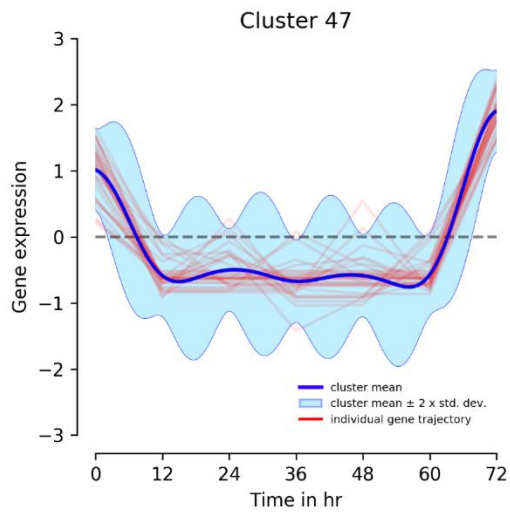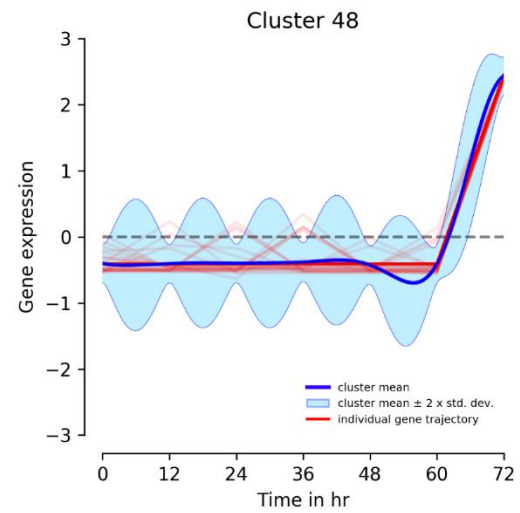

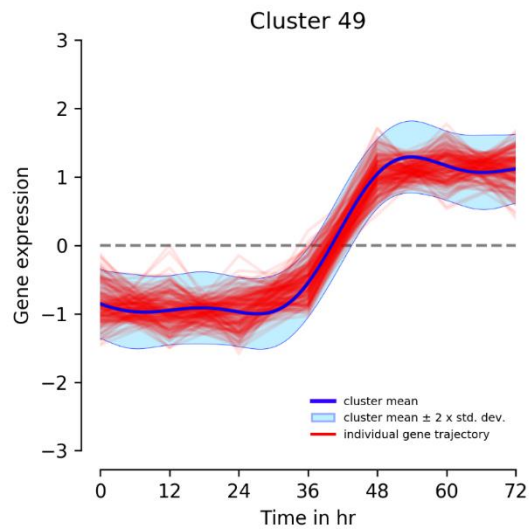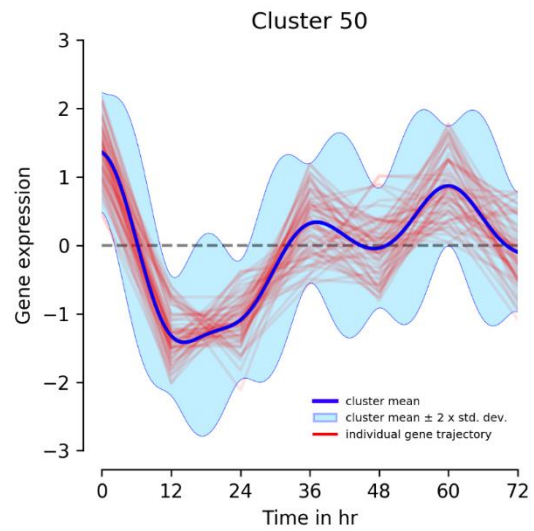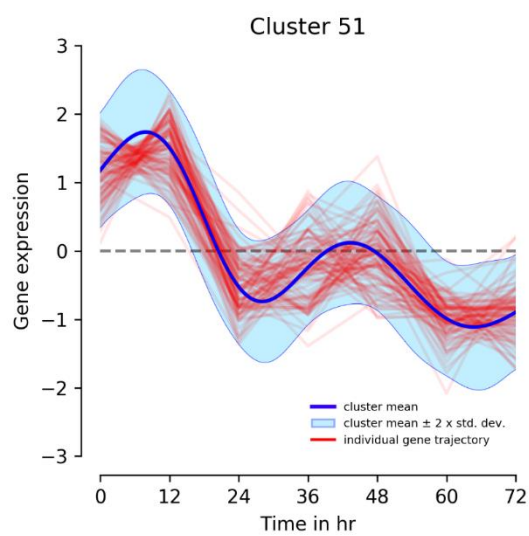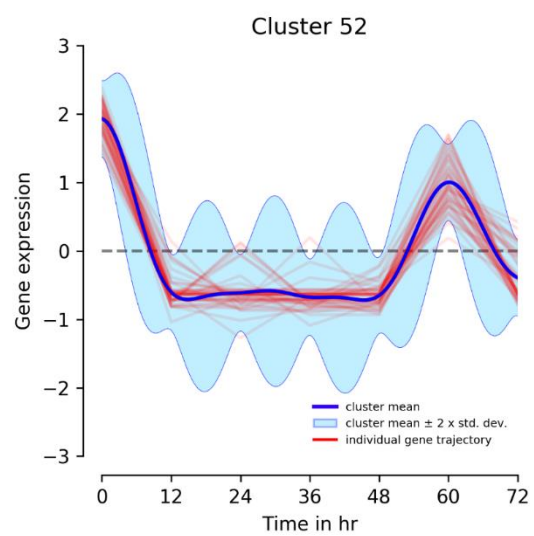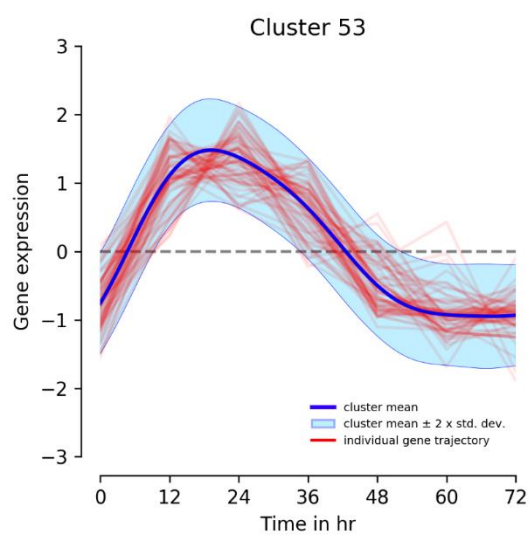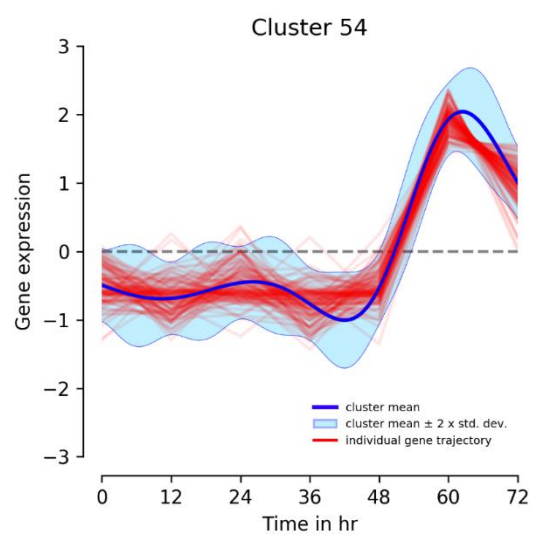

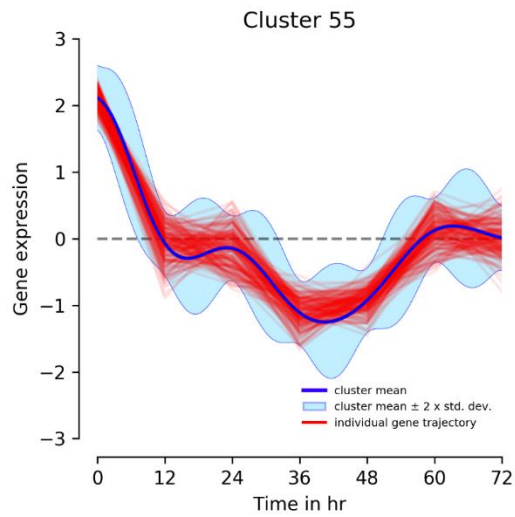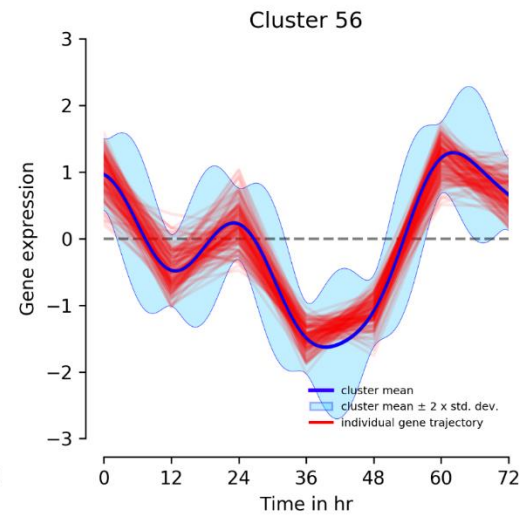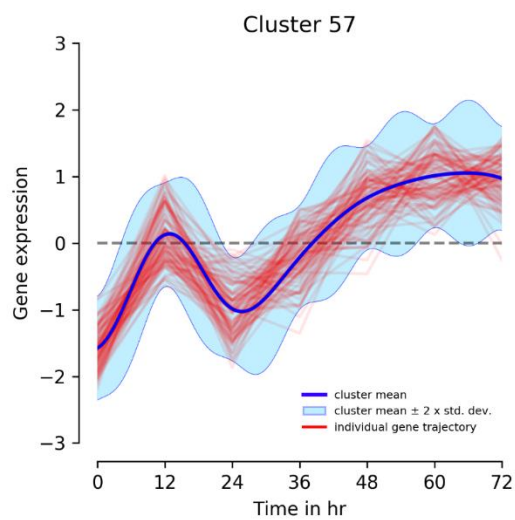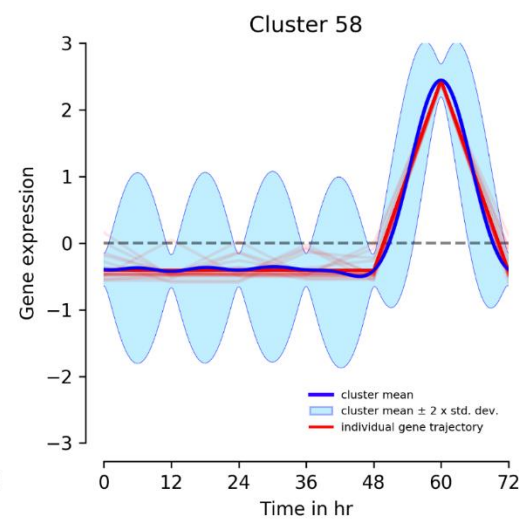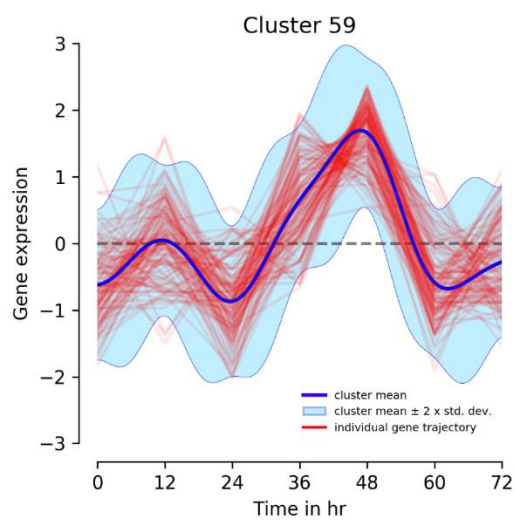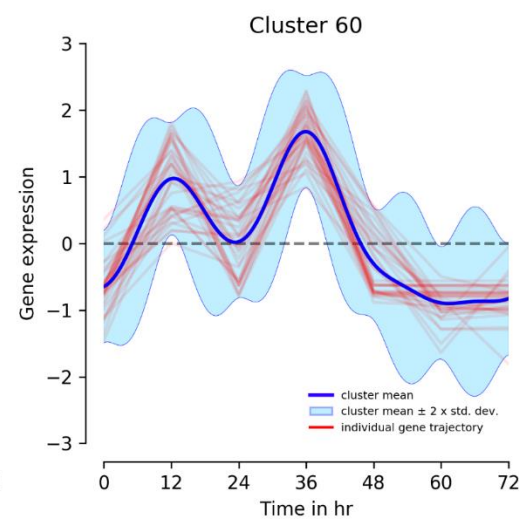

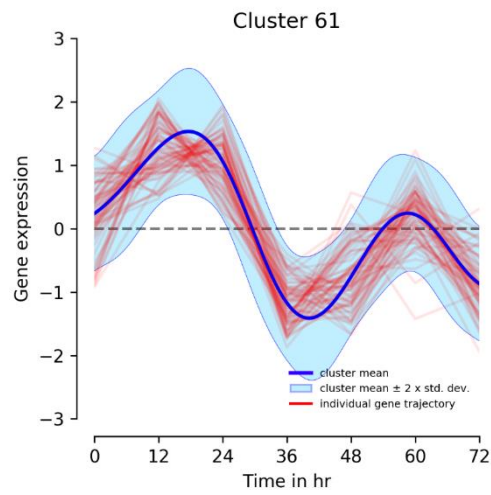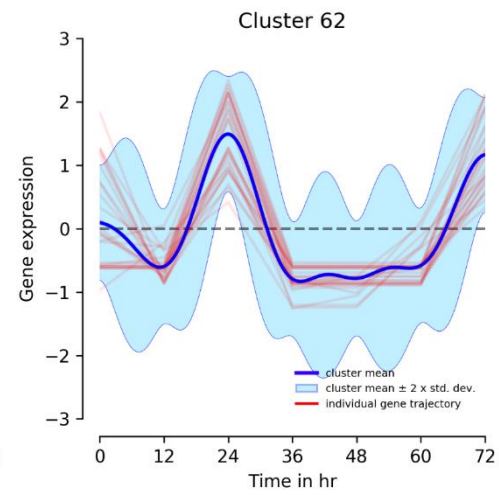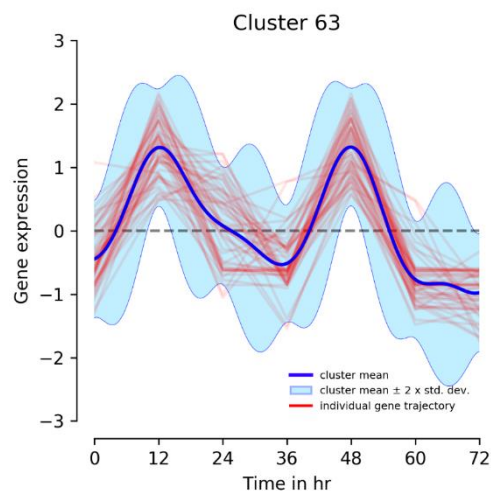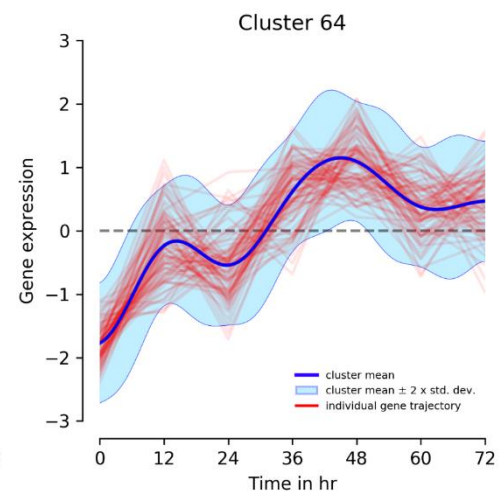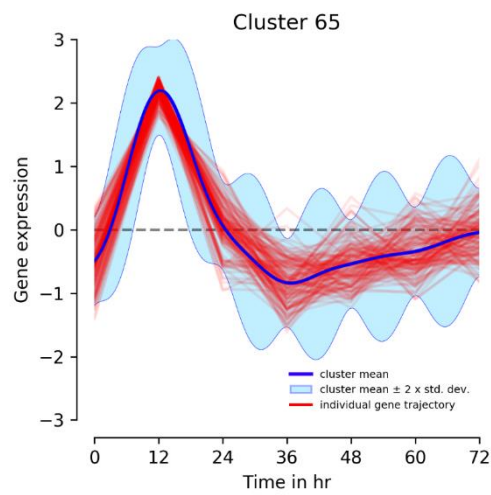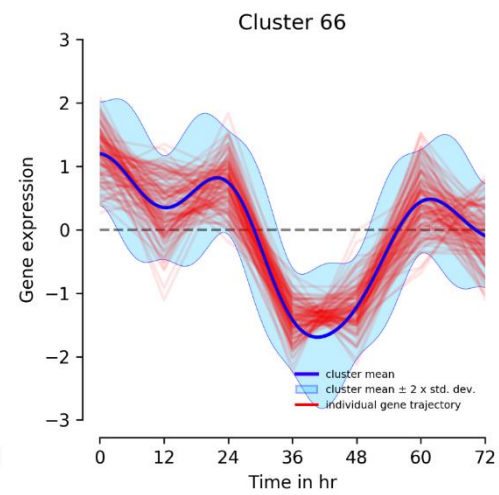

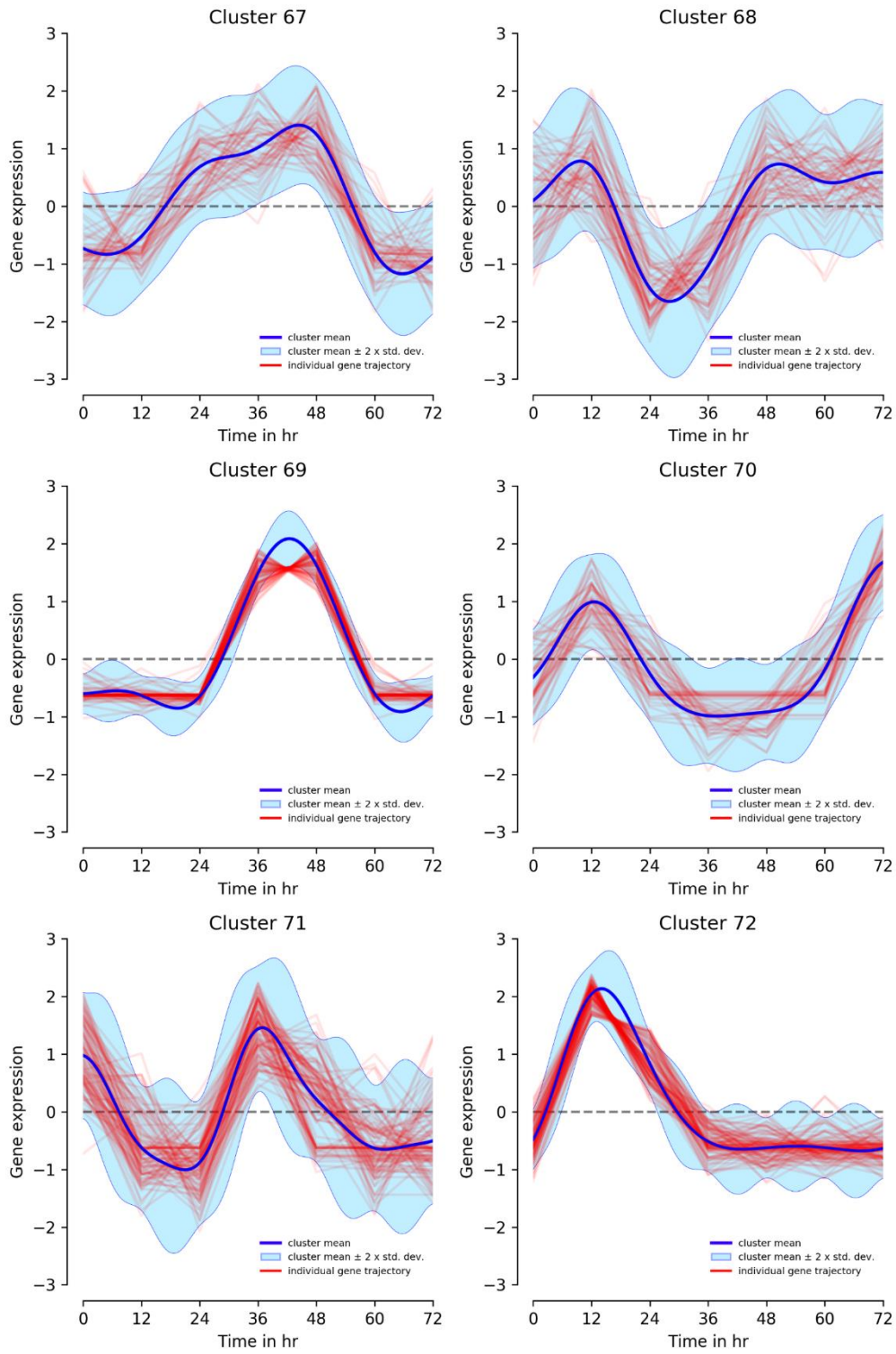

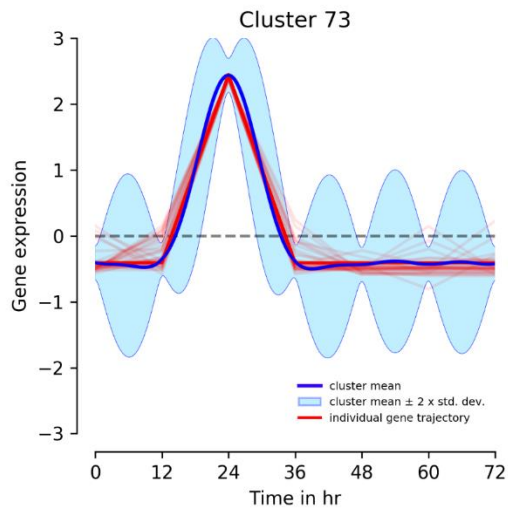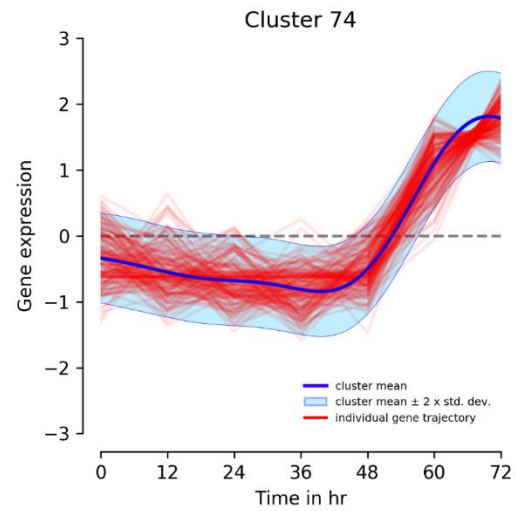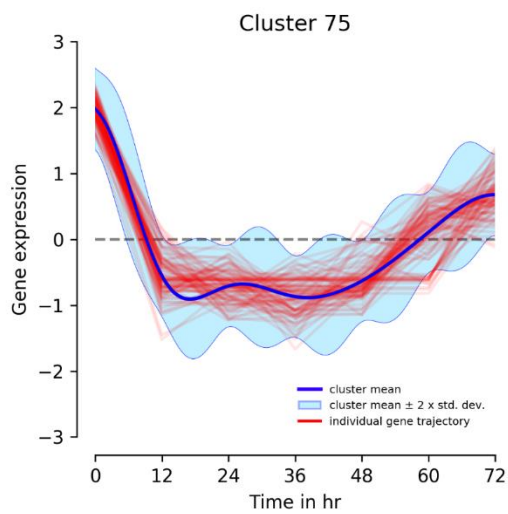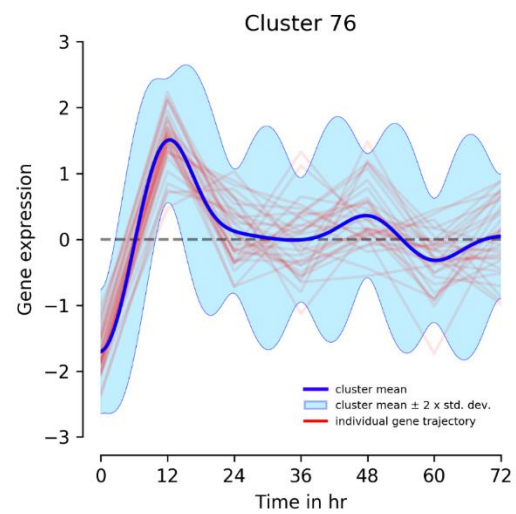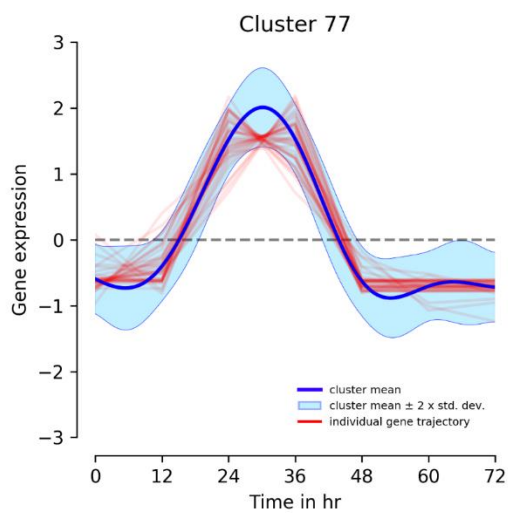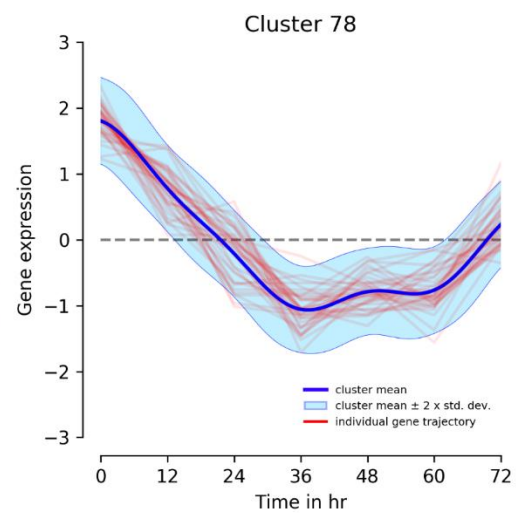

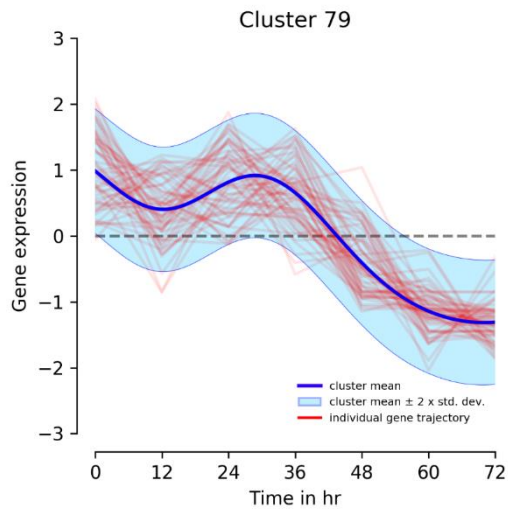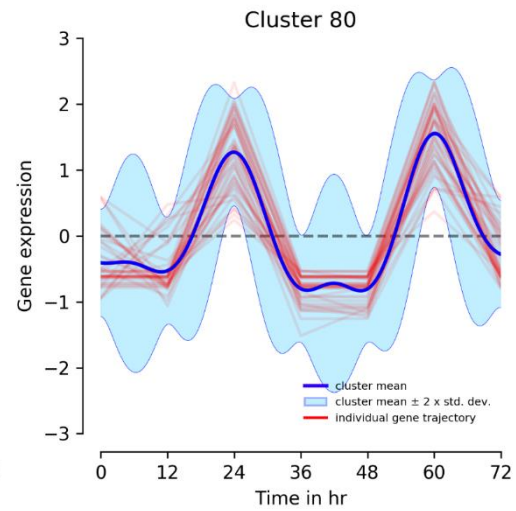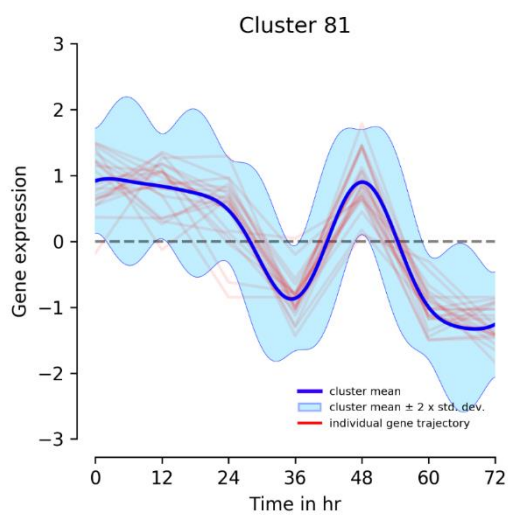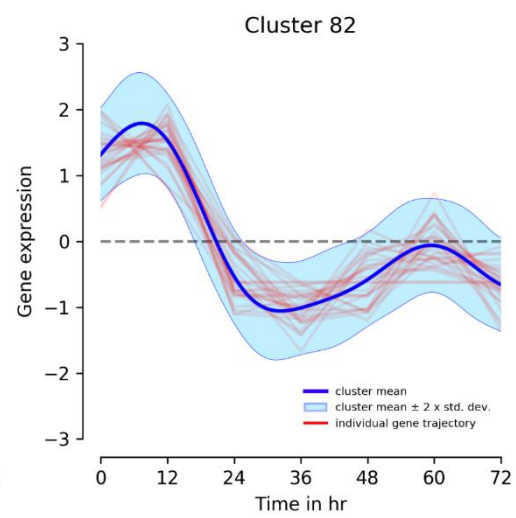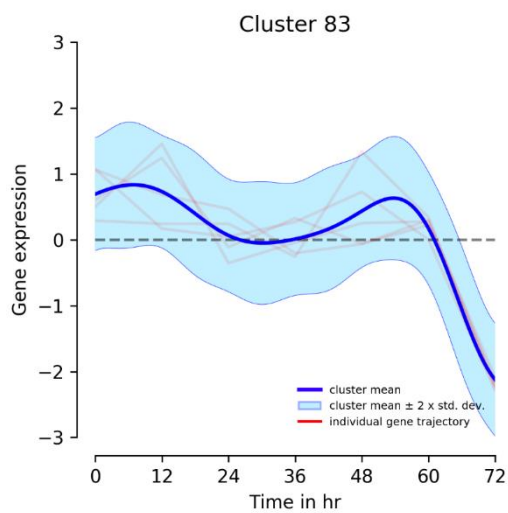

Supplement: Supplementary file 2 [file DataSheet4.PDF]
